# Supplementary material for: Sox9 Inhibits Cochlear Hair Cell Fate by Upregulating Hey1 and HeyL Antagonists of Atoh1
Source: Cells. 2023 Aug 25;12(17):2148. doi: 10.3390/cells12172148 (PMC10486728; doi:10.3390/cells12172148)
Supplement: Supplementary file 1 [file cells-12-02148-s001.zip › cells-2563264-supplementary.pdf]

Supplemental Figure S1 : Sox9 overexpression blocks HC differentiation but does not interfere with SC development. E14.5 cochleae were electroporated with GFP **(A-B)** or Sox9-GFP **(C-P)** and cultured for 6DIV. Explants were immunostained for Myo6 HC marker and Sox2 or p27 SC markers. Orthogonal views of the confocal images (A-D) show that Sox9 transfected cells fail to express HC marker while control GFP transfected cells express mature HC marker. Within the HC layer, some cells that fail to adopt an HC fate in Sox9-GFP electroporated explants were weakly positive for Sox2 marker (white arrows). Still, most of them had lost Sox2 expression (yellow arrowheads), suggesting they did not become SC. **(I-P)** When confocal images were taken at the level of SC, Sox9-GFP transfected cells expressed normal levels of p27 **(I-J and and M-N)** or Sox2 **(K-L and O-P)**, suggesting Sox9 overexpression in cells destined to develop as SC does not affect their differentiation program. Scale bars: 25μm.

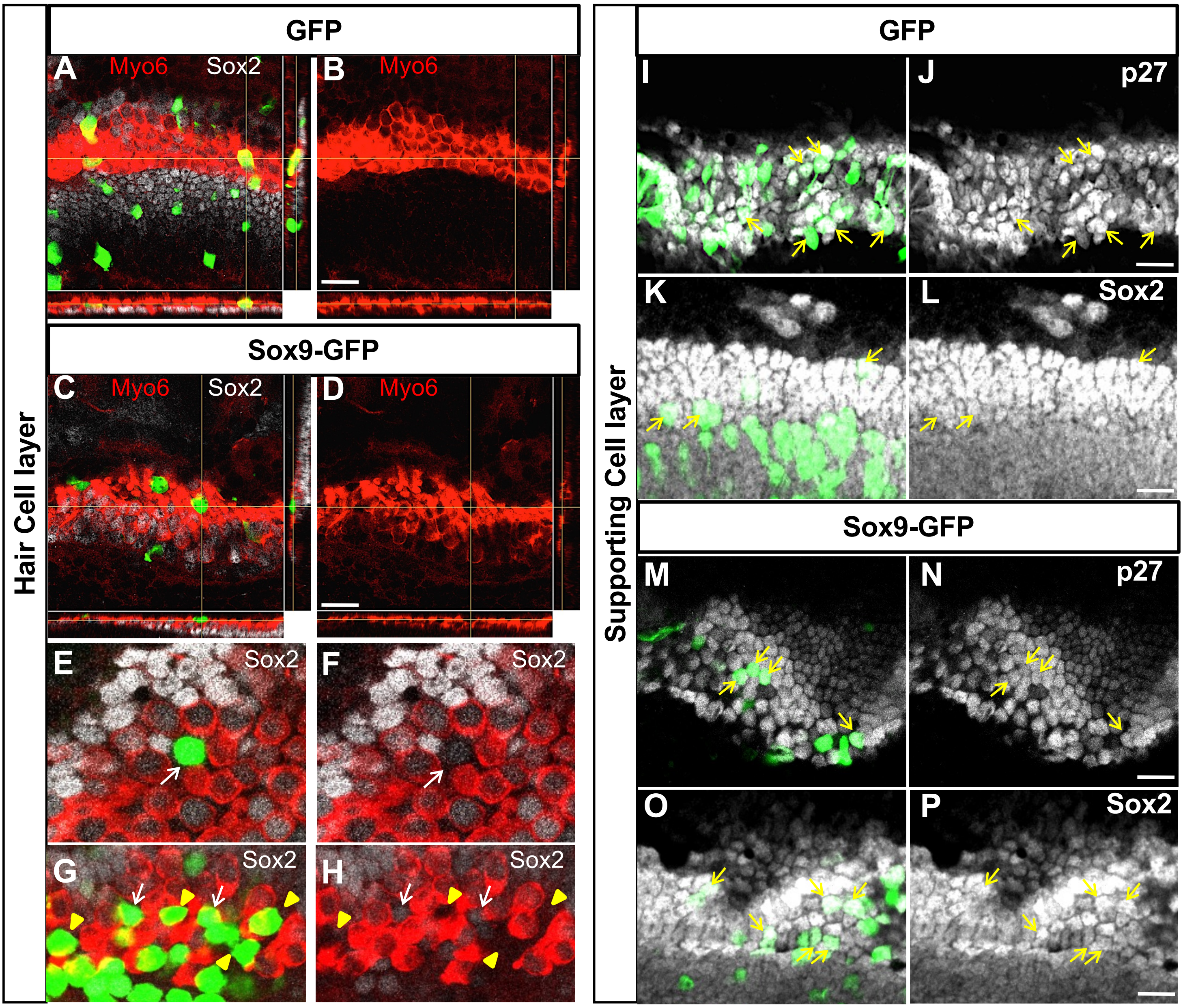

Supplemental Figure S2: Sox9 is efficiently downregulated or inactivated by specific shRNA or DN-Sox9 (A) UB/OC1 cells were transfected with control or Sox9-targeted shRNAs expressing vectors and the level of Sox9 protein was monitored by western blot 48h post-transfection. Actin was used as a loading control. **(B)** UB/OC1 cells were transfected with Col2a1-Luc and pRL-SV reporter vectors and Sox9 or DN-Sox9-expressing plasmids. Two days post-transfection, cells were lysed and assayed for Luciferase and Renilla activities. The Luciferase activities were normalized over Renilla and a representative result (mean ± SD) is presented relative to control transfection. **(C-H)** E14.5 cochleae were electroporated with GFP (C, D), Sox9-GFP (E, F) or Sox9-GFP together with DNSox9-GFP (G, H) and cultured for 6 days before subjected to Myo6 immunostainings. While none of the Sox9-GFP electroporated cells developed as Myo6-positive HCs, a normal ratio of HC was formed when DNSox9 was co-expressed in the explants (yellow arrows). **(I)** Quantifications of C-H: the percentage of GFP-positive cells expressing Myo6 HC marker was evaluated within the sensory epithelium. DNSox9 and Sox9 were co-electroporated in a 2:1 or 4:1 ratio. **(J)** RT-qPCR were performed from UB/OC1 cells transfected with expression vectors for control GFP, Sox9, Sox9 and DNSox9 (ratios of 1:1 and 1:2) or DNSox9 alone. The expression levels were normalized over GAPDH transcript level and reported to control-GFP condition (mean ± SD from 3 individual experiments). The Sox9-induced upregulation of Hey1 and HeyL genes is suppressed by the dominant negative form of Sox9. *** =$p < 0.001$

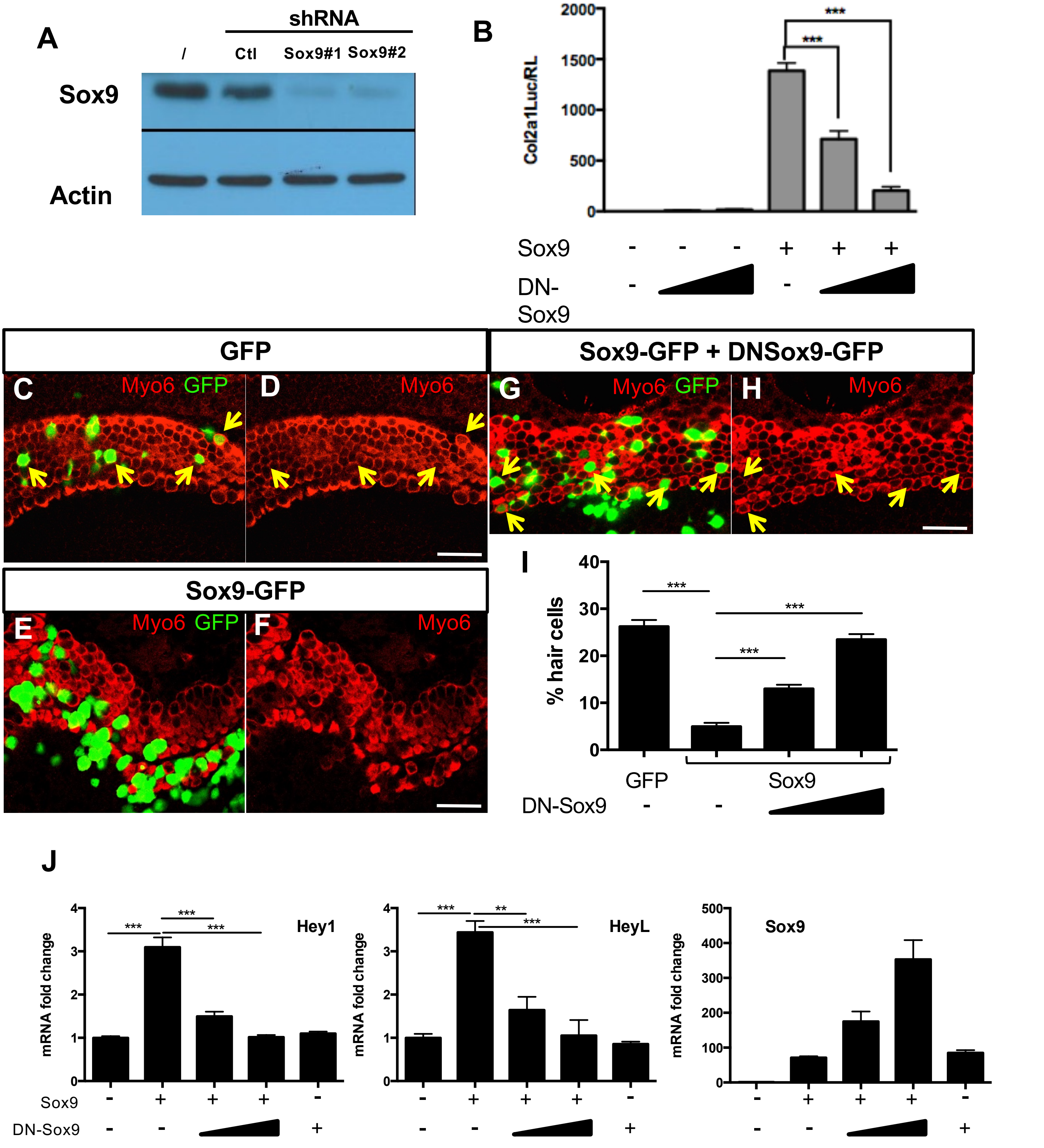

Supplemental Figure S3: Hey1 and HeyL expression pattern in the developing cochlea. Hey1 and HeyL in situ hybridization performed on E14.5, E16.5 and P1 cross-sections of wild-type cochleae. Hey1 is expressed in the sensory epithelium at E14.5, whereas HeyL is weakly expressed at this stage. At E16.5, Hey1 disappears from future HCs following the baso-apical gradient of cellular differentiation, while HeyL is induced in developing SCs. Hey1 and HeyL expression is restricted to supporting cells at P1 (arrowheads indicate inner HCs, brackets indicate outer HCs). Scale bars: 50μm.

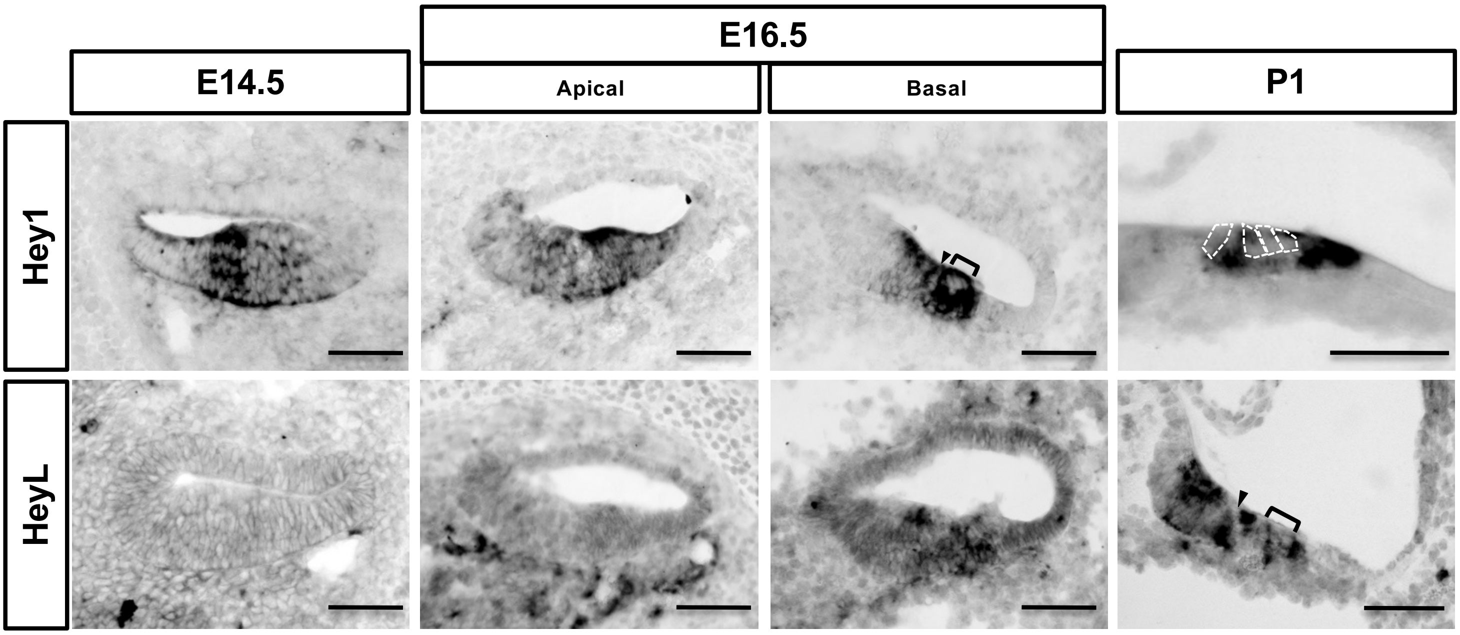

Supplemental Table S1: Hair cell fate in electroporation experiments.
E13.5-E14.5 cochleae were electroporated with shRNA or expression plasmids (IRES-GFP) and cultured for 6 days to allow HC and SC differentiation. The explants were then immunostained for specific HC markers (Myo6 or Parv), and the percentage of GFP+ cells expressing HC marker was evaluated for each explant. Results are indicated as mean ± SEM in the last column of the table. The total number of GFP+ and Myo6+ or Parv+ cells, independently of the number of explants used for quantifications are also indicated.

	Expression vector	Total number of Myo6+ or Parv+/Total number of GFP+	HC (% of GFP+)<br><br>mean ± SEM (n=number of explants)
**Figure 2O**	GFP	37/127	28.50 ± 2.15 % (n=6)
	Sox9-GFP	4/196	1.52 ± 1.13 % (n=11)
	Shctrl-GFP	183/668	26.80 ± 1.75 % (n=10)
	ShSox9#1-GFP	91/254	37.32 ± 2.68 % (n=8)
	ShSox9#2-GFP	87/261	34.95 ± 2.09 % (n=9)
**Figure 3E**	GFP	37/127	28.50 ± 2.15 % (n=6)
	Atoh1-GFP	203/255	86.42 ± 3.65 % (n=6)
	Atoh1-GFP + Sox9-GFP	31/386	10.25 ± 3.17 % (n=12)
**Figure 5G**	GFP	84/368	22.49 ± 1.71 % (n=5)
	Hey1-GFP	7/305	3.07 ± 1.05 % (n=8)
	HeyL-GFP	18/289	6.84 ± 0.75 % (n=6)
**Figure 6B**	Shctrl-GFP	139/500	24.26 ± 1.33 % (n=7)
	Sox9-GFP+Shctrl-GFP	11/465	2.13 ± 0.79 % (n=8)
	Sox9-GFP+ShHey1/L-GFP	38/592	7.79 ± 1.61 % (n=8)
**Figure 7D**	GFP	100/358	27.95 ± 0.544 % (n=5)
	Cre-GFP	263/727	37.43 ± 2.493 % (n=8)

Supplemental Table S2: Primer sequences used in RT-qPCR.
Gene expression was assessed by RT-qPCR with specific forward and reverse primers. In addition, gene accession numbers are indicated.

Gene	Forward primer (5' ➔ 3')	Reverse primer (5' ➔ 3')	Accession number
**Sox9**	AGGAAGCTGGCAGACCAGTA	TCCACGAAGGGTCTCTTCTC	NM_011448.4
**Hes1**	GCGAAGGGCAAGAATAAATG	TGTCTGCCTTCTCTAGCTTGG	NM_008235.2
**Hey1**	AGGTTTTGGCCAGGAAAAGA	AGCAGATCCCTGCTTCTCAA	NM_010423.2
**Hey2**	TGAGAAGACTAGTGCCAACAGC	CCTGTAGCCTGGAGCATCTT	NM_013904.1
**HeyL**	ATAGAGAAACGGCGCAGAGA	ACGGTCATCTGCAAGACCTC	NM_013905.3
**Id1**	GAGTCTGAAGTCGGGACCAC	GATCGTCGGCTGGAACAC	NM_010495.3
**Id2**	CCAGAGACCTGGACAGAACC	GCTCAGAAGGGAATTCAGATG	NM_010496.3
**Id3**	ACTCAGCTTAGCCAGGTGGA	GAGATCACAAGTTCCGGAGTG	NM_008321.2
**Gapdh**	CCCAGCAAGGACACTGAGCAAGAG	CCCCTCCTGTTATTATGGGGGTCTG	NM_001289726.1

Supplemental Table S3: Primer sequences used for promoter cloning into pLUC reporter construct. Promoter fragments from Hey1 and HeyL genes were amplified with expand High-fidelity polymerase using specific primers, named according to their respective position relative to TSS.

**mHey1** (position relative to TSS)	Primer sequence	**hHeyL** (position relative to TSS)	Primer sequence
-95	GTTACCCGGGAGCCGT	-500	GTATCTTCCCTTTTCCTCTCCTTC
+3	CCATCCCTTTCCCACG	-200	CCTATGGAGTTGCTGCTGG
		-150	ACCACGACTAACTTCGACAAACTT
		-80	GTTGACCCTTGCGGAATC
		+3	GAGTCCCCGTCTGTCCCT
